# Hysterosalpingocontrast sonography (HyCoSy): evaluation of the pain perception, side effects and complications

**DOI:** 10.1186/1471-2342-13-28

**Published:** 2013-08-23

**Authors:** Roberto Marci, Immacolata Marcucci, Aurelio Aniceto Marcucci, Nicolina Pacini, Pietro Salacone, Annalisa Sebastianelli, Luisa Caponecchia, Giuseppe Lo Monte, Rocco Rago

**Affiliations:** 1 Department of Morphology, Surgery and Experimental medicine, University of Ferrara, Via Aldo Moro 8, Ferrara, Cona, 44124, Italy; 2 Department of Andrology and Pathophysiology of Reproduction, S. Maria Goretti Hospital, Latina, Italy

**Keywords:** Tubal patency, Female infertility, HyCoSy, Pain perception, Transvaginal sonography

## Abstract

**Background:**

Tubal and uterine cavity diseases commonly compromise female fertility. At the present time, hysteroscopy, laparoscopy with chromopertubation and RX-Hysterosalpingography (RX-HSG) are widely accepted screening procedures enabling the effective assessment of both tubal patency and uterine cavity. Nevertheless, consistent evidence supports the reliability of Hysterosalpingocontrast sonography (HyCoSy) in uterine cavity and tubal patency investigation, as a part of the standard infertility work-up. This prospective study was aimed at evaluating the tolerability of the technique as well as the incidence of related side effects and complications in a large series of infertile patients.

**Methods:**

Pain perception of 632 infertile women was measured by means of an 11-point numeric rating scale. Side effects and late complications were also recorded.

**Results:**

The mean numeric rating scale was 2.15 ± 2.0 SD. Most of the patients (374/632, 59.17%) rated HyCoSy as a non-painful procedure, whereas 24.36% (154/632) women reported mild pelvic pain and 9.96% (63/632) classified the discomfort as “moderate”. Only 6.48% (41/632) of the patient population experienced severe pelvic pain. Fifteen (2.37%) patients required drug administration for pain relief. Twenty-six patients (4.11%) showed mild vaso-vagal reactions that resolved without atropine administration. No severe vaso-vagal reactions or late complications were observed.

**Conclusions:**

HyCoSy is a well-tolerated examination and the associated vagal effects are unusual and generally mild. Consequently, we support its introduction as a first-line procedure for tubal patency and uterine cavity investigation in infertile women.

## Background

**Figure 1:**
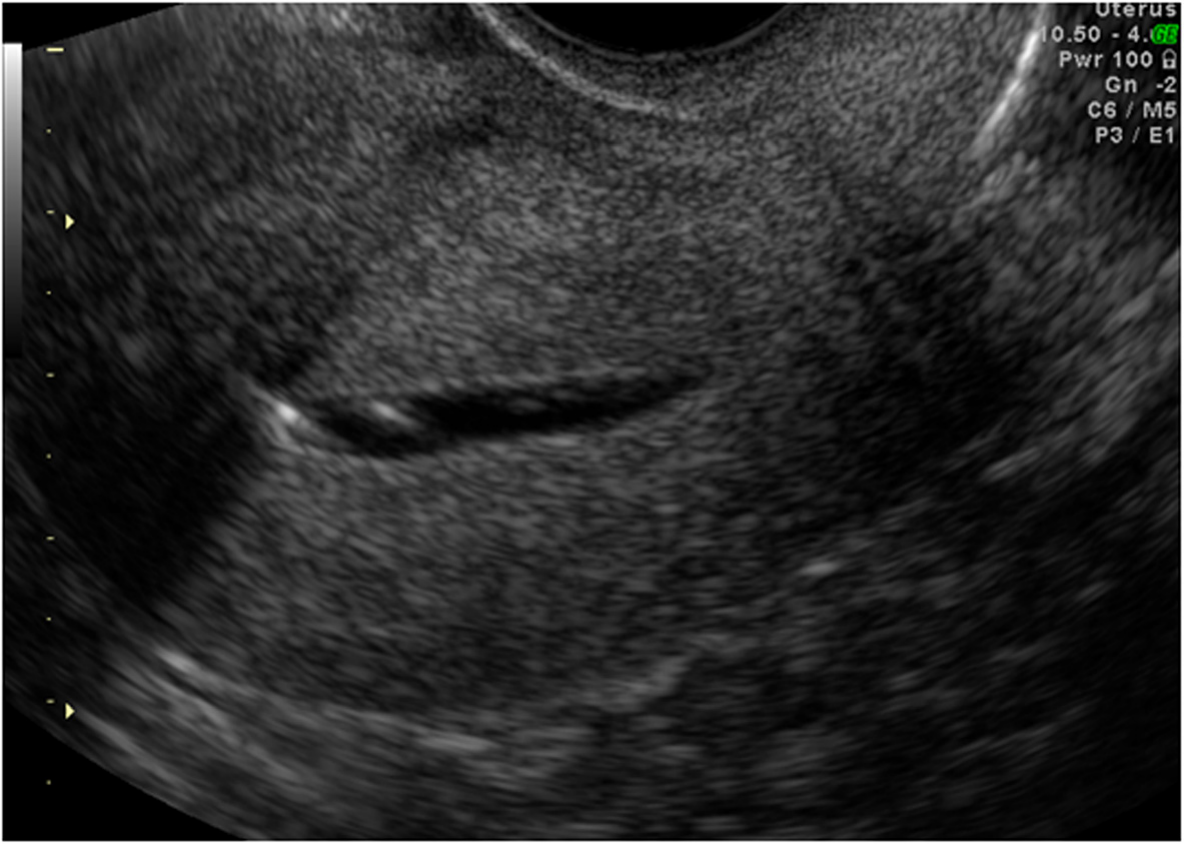
HyCoSy: saline contrast medium expanding a morphologically normal uterine cavity.

**Figure 2:**
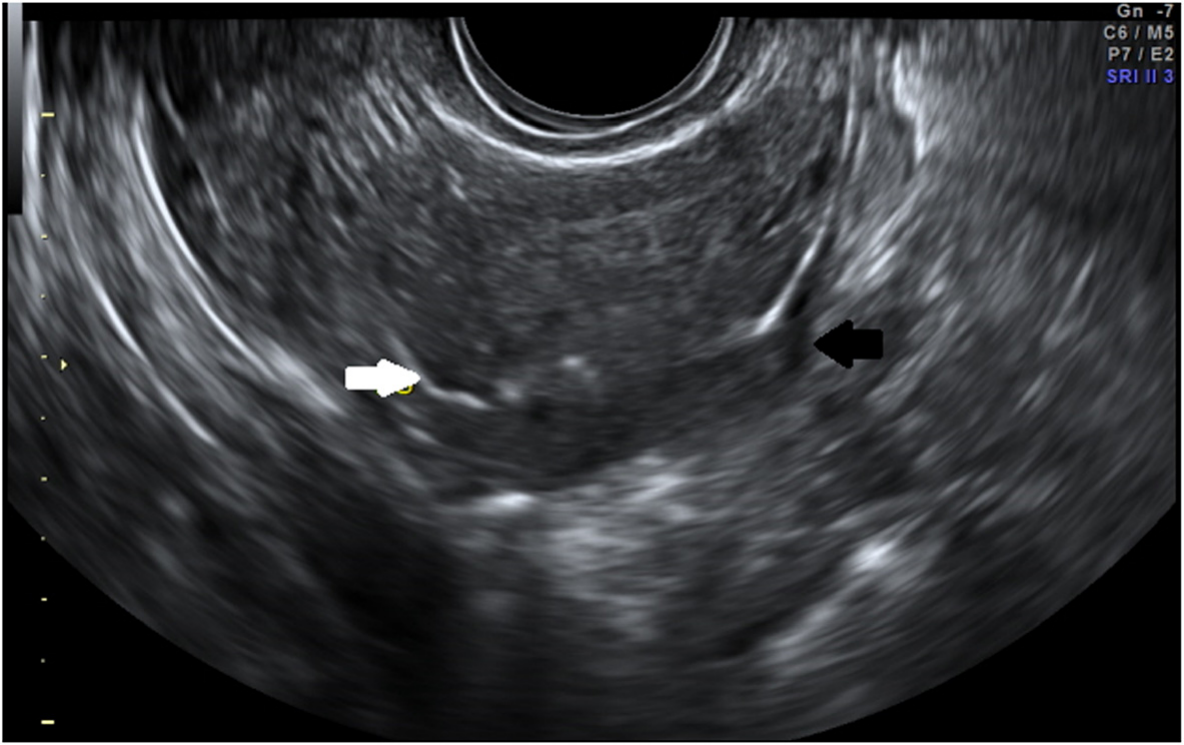
HyCoSy: direct visualization of bilateral tubal patency. (Black arrow: right tube; white arrow: left tube).

**Figure 3:**
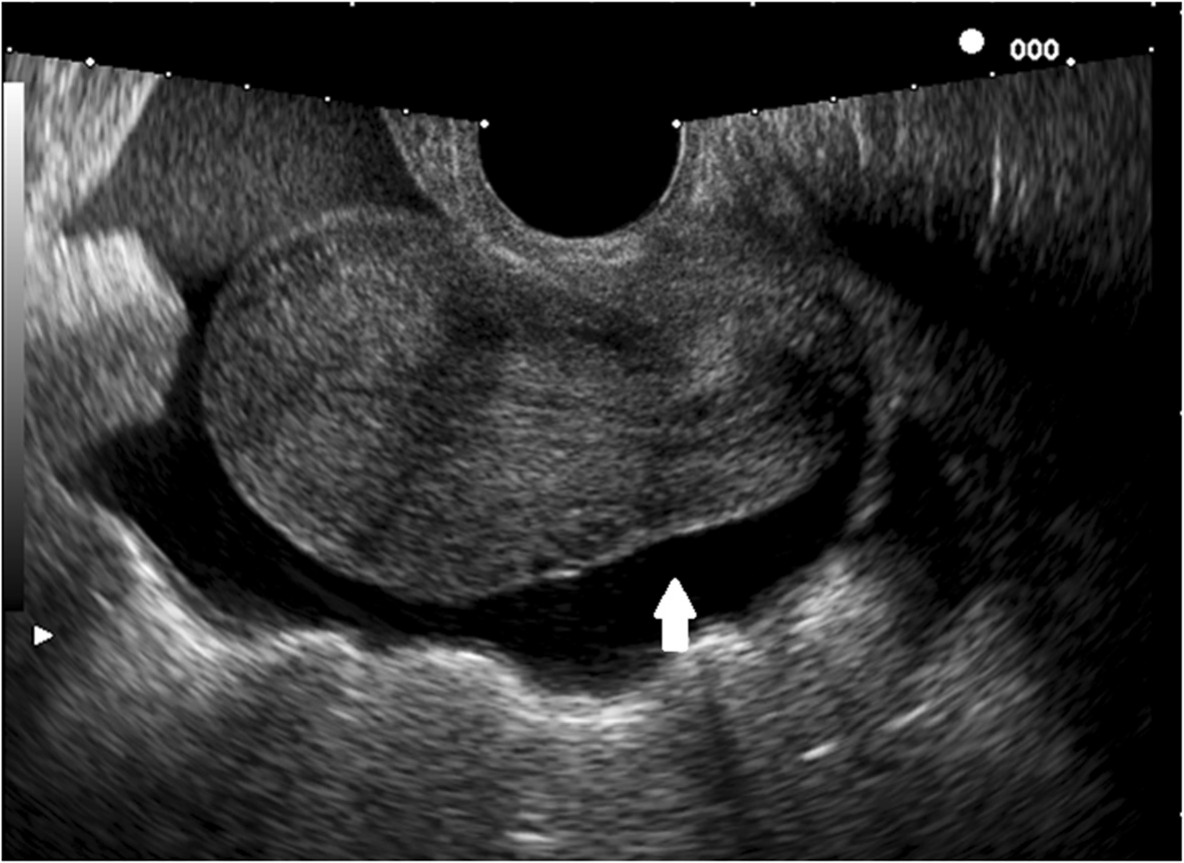
HyCoSy: the periuterine fluid collection (white arrow) is an indirect sign of tubal patency.

The etiology and pathophysiology of infertility are unexplained in some couples, but one-third of infertility cases are related to female factor. Tubal and uterine cavity diseases commonly compromise female fertility (14% of couples who require specialist treatment) [1]. In particular, uterine anomalies or structural abnormalities of the fallopian tubes are diagnosed in 3% and 16% infertile women, respectively [2]. For this reason, tubal and uterine examination plays a major role in the evaluation of the infertile couples and it is mandatory before assisted reproductive techniques (ART) such as intrauterine insemination or in vitro fertilization, is started [3]. At the present time, hysteroscopy [4,5], laparoscopy with chromoperturbation and Rx-Hysterosalpingography (RX-HSG) [6] are widely accepted procedures for the assessment of tubal patency and uterine cavity. However, these techniques show several limitations including: long waiting lists in some hospitals, invasiveness (or minimal invasiveness in the case of office hysteroscopy), painfulness and possible surgical and anesthesiologic risks [7]. In addition, RX-HSG is associated to exposure to ionizing radiations and involves the direct injection of a iodinated contrast agent into the uterine cavity, possibly resulting in atopic phenomena [8]. In the last 10 years, Hysterosalpingocontrast sonography (HyCoSy) has been introduced in clinical practice as an effective tool for tubal patency and uterine cavity evaluation (Figures 1, 2 and 3). This investigation is considered safe, well tolerated, rapid, easy to perform and inexpensive [9,10]. HyCoSy is a transvaginal sonography in which a galactose solution containing galactose microbubbles or an inexpensive mixture of air and saline solution is injected into the uterine cavity using a cervical catheter. Several studies [6,9-19] have shown that HyCoSy displays high specificity and sensitivity in tubal patency and uterine cavity assessment. On the contrary, only limited evidence is available on both the tolerability and the real incidence of side effects and complications related to this procedure. This prospective study was aimed at evaluating these parameters in a large series of infertile patients undergoing HyCoSy for tubal patency and the uterine cavity assessment.

## Methods

Six hundred thirty-two infertile women, who referred to our clinic between January 2008 and November 2012, were consecutively enrolled. Patients underwent HyCoSy at the Andrology and Pathophysiology of Reproduction Unit of S. Maria Goretti Hospital in Latina. This work has been carried out in accordance with The Code of Ethics of the World Medical Association (Declaration of Helsinki) for experiments involving humans. Approval from the institution’s ethics committee has been obtained before starting the study. The aim of the examination as well as the possible side effects and complications associated to the procedure were explained to the patients and informed consent was provided in each case before starting the procedure. The clinical data of the study group are summarized in Table 1. Exclusion criteria were a history of infertility lasting less than one year, abnormal bleeding, active pelvic infections and uterine malignancies. We performed pelvic examination, Pap smearing test and transvaginal ultrasound before HyCoSy. Vaginal and cervical swabs as well as blood samples for hormonal profile and serologic markers were also taken. Women did not receive any pain medication or antibiotic treatment before undergoing the procedure. The patients were examined in the lithotomic position, during the first phase of the menstrual cycle (day 9–11 of menstrual cycle). Patients were asked to use contraceptives or avoid sexual intercourse from the last menstrual period until the day of the exam. A preliminary transvaginal ultrasound was performed by two skilled operators with a 7 MHz probe (Logiq 5 Expert GE, GE Healthcare, United Kingdom) in order to exclude any uterine or adnexal pathology and to localize the ovaries and the interstitial part of the salpinges before the injection of the ultrasound contrast medium. The vulva and the cervix were previously disinfected with chlorhexidine. A 5 F stylet catheter (CooperSurgical, Germany) was placed into the cervical os under direct visualization and the balloon was filled with 1–1.5 ml of saline solution to fix the catheter and prevent saline backflow. Then, saline solution (0.9% sodium chloride) was slowly injected. The uterine cavity was evaluated on transverse and longitudinal images. The operator subsequently injected into the catheter a total volume of 10 to 20 mL saline solution alternated to little boluses of air. Air bubbles, that are highly echogenic, would facilitate checking the patency of the fallopian tubes. The patency of a tube was determined by the passage of air bubbles through the tube and/or the presence of liquid and air in the abdominal cavity near the ovarian fossas. At the end of the procedure, the patients were monitored for about 15 minutes in supine position in order to prevent the onset of vasovagal reactions or scapular pain, due to the irritation of the phrenic nerve. After the execution of the HyCoSy, all participants were asked about the pain (type, location, duration, irradiation) they experienced. In order to quantify pain perception during the procedure we used an 11-point (0 to 10) numerical rating scale, on which 0 corresponded to no pain at all and 10 indicated severe pain. The patients were familiarized with the scale before the procedure was performed. In accordance to Savelli et al. [20], pelvic pain was classified as “absent”, “mild”, “moderate” and “severe” when rated as 0, 1 to 4, 5 to 7 and 8 to 10, respectively.

**Table 1:** Clinical features of the patients who underwent HyCoSy

	**N (%)**
N. of patients	632
Mean age (years)	33.2 ± 5.4 SD
	(range 22–44)
Mean infertility duration (months)	60.4 ± 31.2 SD
	(range 24–96)
Primary infertility	452/632 (71.5%)
Secondary infertility	180/632 (28.5%)
Associated pelvic diseases	191/632 (30.2%)
• Myomas	• 95/632 (15%)
• Pelvic endometriosis	• 54/632 (8.5%)
• Uterine congenital malformations	• 37/632 (5.8%)
• Endometrial polyps	• 5/632 (0.8%)
Tubal patency:	
• Monolateral	503/632 (79.5%)
• Bilateral	53/632 (8.3%)
• Bilateral occlusion	76/632 (12%)

## Results

**Figure 4:**
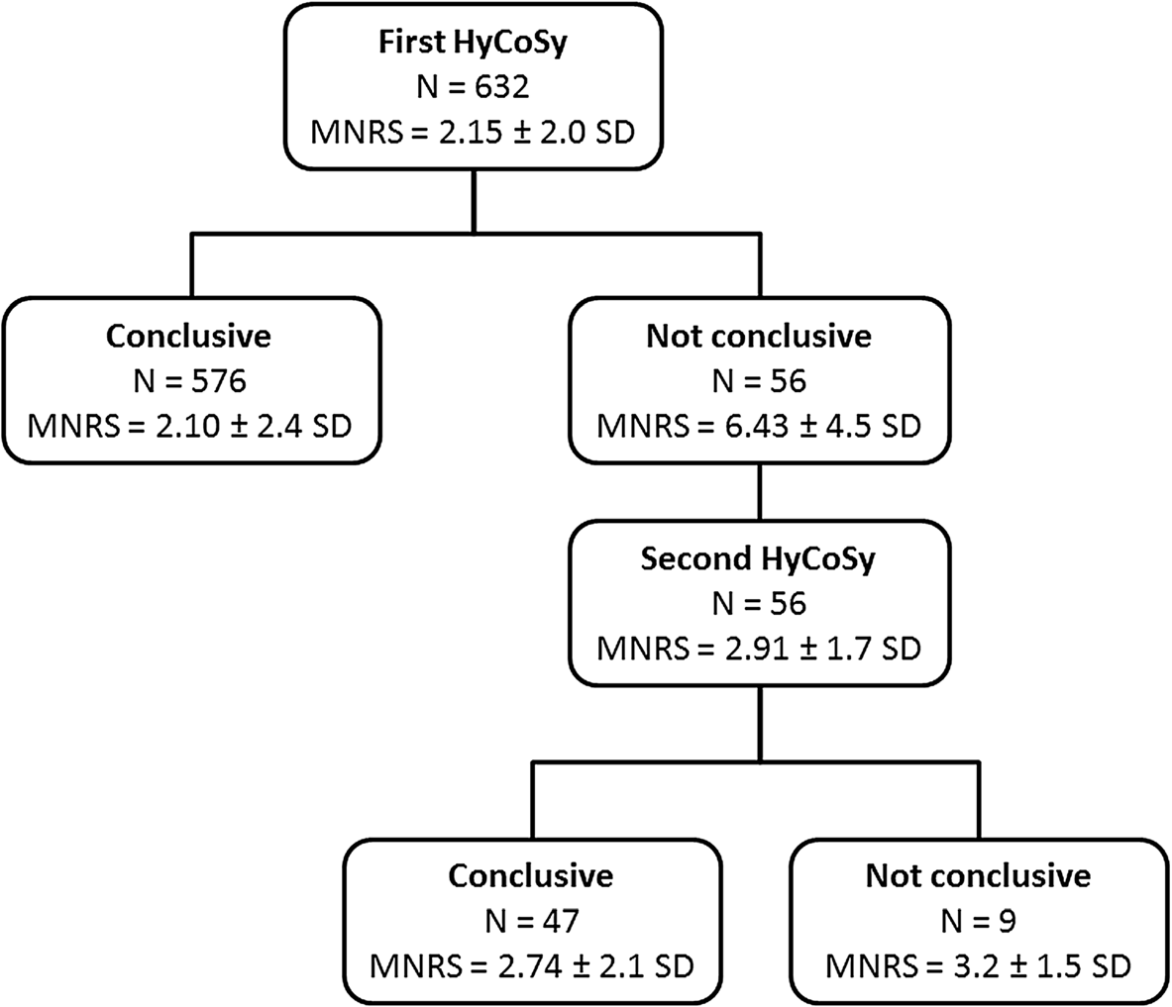
Flowchart representing the distribution of women who repeated HyCoSy twice (MNRS, mean numeric rating scale; SD, standard deviation).

The mean overall numeric rating scale was 2.15 ± 2.0 SD. The duration of the procedure, from the insertion to the extraction of the catheter, ranged from 10 to 20 minutes. The majority of patients (374/632; 59.17%) considered HyCoSy a non-painful procedure, whereas 24.36% (154/632) reported mild pelvic pain and 9.96% (63/632) classified the discomfort as “moderate”. Only 6.48% (41/632) of the population experienced severe pelvic pain. After HyCoSy, 15 (2.37%) patients required drug administration for pain relief. Twenty-six patients (4.11%) showed mild vaso-vagal reactions (pallor, nausea, sudation, hypotension, bradycardia) that resolved without atropine administration. No severe vaso-vagal reactions (vomiting, confusion, syncope) and late complications (haemorrhage, pelvic inflammatory disease (PID), fever) were reported. We diagnosed bilateral tubal patency in 79.5% (503/632) women, unilateral patency in 8.3% (53/632) and bilateral tubal occlusion in 12.02% (76/632). In 56/632 (8.8%) patients HyCoSy was not conclusive and the exam was repeated during the subsequent menstrual cycle. Women who underwent examination for the second time considered HyCoSy less painful than the first time (mean overall numeric rating scale 2.9 vs 6.4) (Figure 4). The second HyCoSy revealed bilateral tubal patency in 62.5% (35/56) cases and monolateral tubal patency in 21.4% (12/56), whilst diagnostic laparoscopy or RX-HSG were needed to set a definitive diagnosis in the remaining 16% (9/56) cases. In 191/632 (30%) cases we demonstrated an associated pelvic disease. The clinical features of the patient population as well as the main results of the study are summarized in Table 1 and Table 2.

**Table 2:** Pain perception, side effects and complications

	**N (%)**
Mean numeric rating scale	2.15 ± 2.0 SD
Pain perception:	
• Absent	374/632 (59.17%)
• Mild	154/632 (24.36%)
• Moderate	63/632 (9.96%)
• Severe	41/632 (6.49%)
Painkiller required	15/632 (2.37%)
Mild vaso-vagal reactions (pallor, nausea, sudation, hypotension, bradycardia)	26/632 (4.11%)
Severe vaso-vagal reactions (vomiting, confusion, syncope)	0/632 (0%)
Complications (haemorrhage, PID, fever)	0/632 (0%)

## Discussion

According to several studies, HyCoSy shows high overall accuracy in the evaluation of both tubal patency and uterine cavity morphology [6-19]. Furthermore, HyCoSy avoids both exposure to ionizing radiation and injection of iodinated contrast medium that could potentially result toxic. In addition, HyCoSy is inexpensive, fast and devoid of surgical and anestesthesiologic risks, as opposed to laparoscopy with chromopertubation and hysteroscopy [7,8]. As other diagnostic methods, HyCoSy does not always provide exhaustive information about tubal and uterine cavity morphology [16]; in particular, it could display misleading images in case of distal tubal obstruction or complicated pelvic diseases (i.e. PID, endometriosis, previous appendicitis, abdomino-pelvic inflammation) [15]. In such doubtful clinical conditions, laparoscopy with chromoperturbation and hysteroscopy should still be considered the “gold standard” for tubal and uterine cavity assessment, respectively.

**Figure 5:**
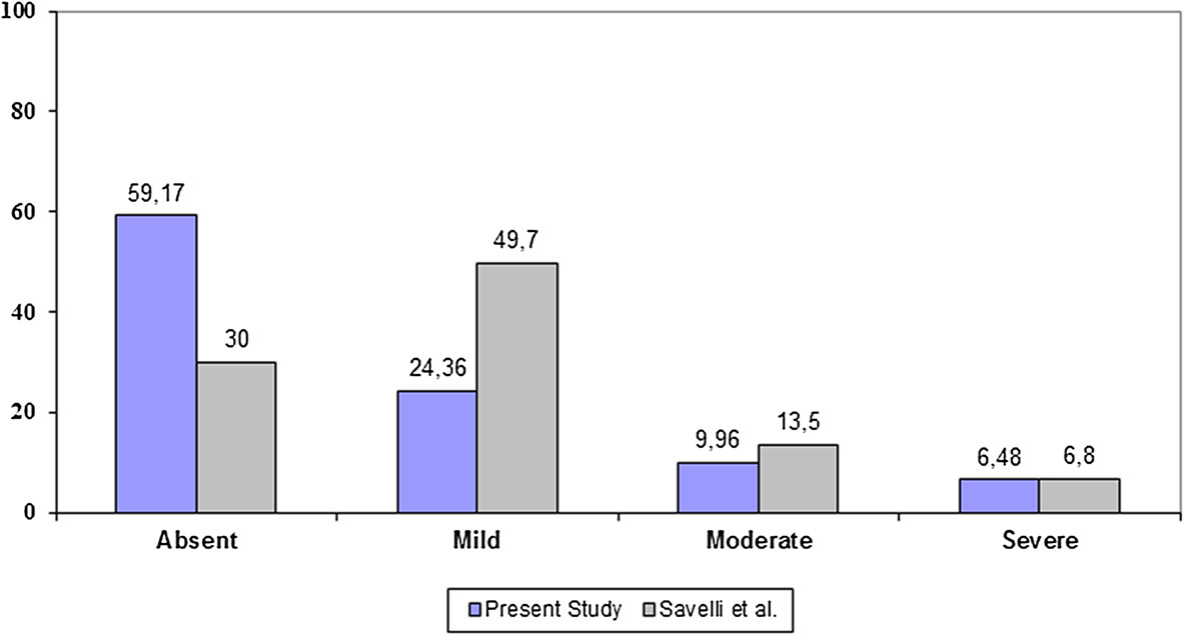
Evaluation of pain perception in our series and data reported by Savelli et al., 2009.

Our data prove that HyCoSy is a well-tolerated examination and it is associated to a low incidence of related complications and side-effects. The pain experienced by the patients is usually mild and comparable to the cramping pain felt during a normal menstrual cycle. Similar findings are reported by Savelli et al. in a prospective study [20]. The Authors evaluated pain perception by means of an 11-point numeric rating scale in 669 infertile women undergoing HyCoSy. The mean numeric rating scale was 2.7 ± 2.5, only 2% of patients required a painkiller for pain relief and mild or severe vasovagal reactions were observed in 4.1% and 0.8% cases, respectively. Our data concerning pain experience during HyCoSy are compared to those provided by Savelli et al. in Figure 5. In both studies only few patients reported moderate/severe pain. The only difference concerns the number of patients who felt no pain at all or mild discomfort. However, we observed that several women were frequently unable to distinguish clearly whether the sensation experienced during the examination, and in particular after the inflation of the balloon into the cervical canal, was slightly painful or just a nuisance comparable to the insertion of the vaginal speculum. As a result, we believe that the findings of the two studies are similar.

In another recent study Graziano et al. evaluated efficacy, compliance and cost effectiveness of HyCoSy, hysteroscopy, and RX-HSG for uterine cavity evaluation and tubal patency determination [21]. HyCoSy showed high sensitivity, specificity, positive predictive values (PPV) and negative predictive values (NPV) and did not display any significant difference when compared to hysteroscopy. Additionally, HyCoSy proved to be as accurate as RX-HSG in detecting a monolateral or bilateral tubal obstruction. Finally HyCoSy was associated to milder pain perception and lower costs when compared to RX-HSG and hysteroscopy. In particular, the Authors assessed pain by means of an 8-point (0 to 7) numerical rating scale on which 0 corresponded to no pain at all and 7 indicated severe pain. Pelvic pain was classified as “absent”, “mild”, “moderate” and “severe” when rated as 0–1, 2–3, 4–5 and 6–7, respectively. An “absent-mild” discomfort was reported in 80.84% women undergoing HyCoSy, in 52.9% women undergoing hysteroscopy and in 9.1% patients undergoing RX-HSG. Ayida et al. compared HyCoSy to conventional RX-HSG as to the tolerability of the procedure [8]. Sixty-six subfertile women underwent one of the two screening procedures, all performed by the same operator. No significant difference in reported procedure time, amount of contrast medium used, patient tolerability or adverse effects was found. In a recent study by Socolov et al., the pain experience of 121 infertile women who underwent both HyCoSy and RX-HSG was analysed [10]. Pain perception was measured using a self-assessment questionnaire. On the basis of the results obtained, HyCoSy was suggested to be a well-tolerated diagnostic procedure, associated with few or mild vagal effects. During HyCoSy pain was rated as slightly higher than during RX-HSG. The correlation between pain perception and other independent variables pertaining to the HyCoSy procedure (i.e. a difficult catheter passage, amount of contrast medium injected, unilateral or bilateral tubal blockage, presence of IgG antibodies to Chlamydia, menstrual cycle phase) was also investigated **in the afore-mentioned study**. The only strong association found concerned the volume of injected contrast medium. The higher volume of contrast medium injected during HyCoSy seems to explain the significantly higher pain level reported.

Pain perception during HyCoSy could be due to uterine distension after saline solution infusion. The mechanical distension of the uterine walls could cause the release of local prostaglandins, resulting in uterine cramps [22]. Although convincing, this hypothesis has been disconfirmed by a recent study. In fact it was demonstrated that a preventive administration of antispasmodic drug such as hyoscine-N-butylbromide, a muscarinic receptor antagonist with anticholinergic effects, does not decrease pain during HyCoSy by affecting uterine contractions [23].

In addition, HyCoSy could also play a therapeutic role in subfertile women. In our infertility centre we noticed that spontaneous pregnancies often occurred in patients who underwent HyCoSy in the previous 3–6 months [24]. Lindborg et al. [25], recently suggested a valid explanation for this phenomenon: the passage of the fluid through a partially occluded tube (minor adhesions, mucus plugs) could remove minor adhesions or buildup material caused by inflammatory processes and hence restore tubal patency.

## Conclusions

HyCoSy is a well-tolerated procedure and the associated vagal effects are unusual and generally mild. It is a safe, efficient and non-invasive diagnostic examination. Furthermore, it provides useful information for the management of infertile couples. Therefore we support its use as a first-line procedure for the evaluation of both the uterine cavity and the tubal patency in infertile women.

## Abbreviations

(HyCoSy): Hysterosonocontrast sonography; (RX-HSG): RX-hysterosalpingography; (ART): Assisted reproductive techniques; (PID): Pelvic inflammatory disease.

## Competing interests

Authors declare to have no commercial and/or financial interest with manufacturers of pharmaceuticals, laboratory supplies, and/or medical devices; they declare to have no relationship with commercial providers of medically related services.

## Authors’ contributions

RM has substantially contributed to design, preparation, drafting and revising the intellectual content of the final version of the manuscript. IM has substantially contributed to data collection, to the diagnostic process, to the preoperative and operative work up linked to the condition of the patient, to preparation, drafting and revising the final version of the manuscript. He also gave an extremely important intellectual support. AAM has substantially contributed to data collection, to the diagnostic process, to the preoperative and operative work up linked to the condition of the patient. NP has substantially contributed to data collection, to the diagnostic process, to the preoperative and operative work up linked to the condition of the patient. PS has substantially contributed to data collection, to the diagnostic process, to the preoperative and operative work up linked to the condition of the patient. AS has substantially contributed to data collection, to the diagnostic process, to the preoperative and operative work up linked to the condition of the patient. LC has substantially contributed to data collection, to the diagnostic process, to the preoperative and operative work up linked to the condition of the patient. GL has substantially contributed to design, preparation, drafting and revising the intellectual content of the final version of the manuscript. RR has substantially contributed to data collection, to the diagnostic process, to the preoperative and operative work up linked to the condition of the patient, to preparation, drafting and revising the final version of the manuscript. He also gave an extremely important intellectual support. All authors read and approved the final manuscript.

## Pre-publication history

The pre-publication history for this paper can be accessed here:

http://www.biomedcentral.com/1471-2342/13/28/prepub
